# Supplementary material for: Deletion of wfs1 Impairs Oligodendrocyte Precursor Cells Dorsal Distribution and Myelination Through the wfs1-hmgcs1 Axis in Zebrafish
Source: Biology (Basel). 2026 Aug 16;15(16):1402. doi: 10.3390/biology15161402 (PMC13510151; doi:10.3390/biology15161402)
Supplement: Supplementary file 1 [file biology-15-01402-s001.zip › File S1-Transcriptome sequencing data and differentially expressed genes (DEGs) in wfs1-- zebrafish at 5 dpf/2.KEGG/wfs1KOvsWT/GSEA/WT/MTOR_SIGNALING_PATHWAY/MTOR_SIGNALING_PATHWAY(DRE04150).html]

Details for gene set MTOR\_SIGNALING\_PATHWAY(DRE04150)[GSEA]

|  || Dataset | deg.sample |
| Phenotype | sample.cls |
| Upregulated in class | WT |
| GeneSet | MTOR\_SIGNALING\_PATHWAY(DRE04150) |
| Enrichment Score (ES) | -0.26600936 |
| Normalized Enrichment Score (NES) | -1.2835314 |
| Nominal p-value | 0.06882591 |
| FDR q-value | 0.55203855 |
| FWER p-Value | 0.987 |
Table: GSEA Results Summary

  

Fig 1: Enrichment plot: MTOR\_SIGNALING\_PATHWAY(DRE04150)      
 Profile of the Running ES Score & Positions of GeneSet Members on the Rank Ordered List

  

| PROBE | DESCRIPTION (from dataset) | GENE SYMBOL | GENE\_TITLE | RANK IN GENE LIST | RANK METRIC SCORE | RUNNING ES | CORE ENRICHMENT || 1 | 79380 | eif4ea |  |  | 35 | 4.922 | 0.0265 | No |
| 2 | 324140\_1 | sgk1 |  |  | 109 | 4.683 | 0.0504 | No |
| 3 | 359840 | atp6v1b2 |  |  | 274 | 3.761 | 0.0663 | No |
| 4 | 796322 | slc3a2a |  |  | 483 | 3.041 | 0.0768 | No |
| 5 | 100006675 | prkcba |  |  | 711 | 2.563 | 0.0840 | No |
| 6 | 100000252\_3 | pik3r3b |  |  | 716 | 2.550 | 0.0981 | No |
| 7 | 553214\_1 | eif4ba |  |  | 877 | 2.272 | 0.1058 | No |
| 8 | 436718 | rhocb |  |  | 948 | 2.171 | 0.1158 | No |
| 9 | 58072\_1 | fzd8b |  |  | 1186 | 1.912 | 0.1190 | No |
| 10 | 245702 | igf1rb |  |  | 1196 | 1.904 | 0.1294 | No |
| 11 | 556087 | wnt2bb |  |  | 1255 | 1.847 | 0.1379 | No |
| 12 | 799099\_1 | lpin1 |  |  | 1367 | 1.749 | 0.1442 | No |
| 13 | 100330617\_1 | tsc1b |  |  | 1449 | 1.691 | 0.1511 | No |
| 14 | 555584\_1 | prr5a |  |  | 1556 | 1.619 | 0.1568 | No |
| 15 | 446167 | grb10a |  |  | 1752 | 1.504 | 0.1591 | No |
| 16 | 100000252 | pik3r3b |  |  | 1965 | 1.382 | 0.1601 | No |
| 17 | 573492\_1 | rragca |  |  | 2319 | 1.241 | 0.1559 | No |
| 18 | 58023\_1 | fzd9b |  |  | 2350 | 1.227 | 0.1619 | No |
| 19 | 100009657\_1 | rps6ka3b |  |  | 2364 | 1.223 | 0.1683 | No |
| 20 | 563560 | ikbkb |  |  | 2417 | 1.202 | 0.1734 | No |
| 21 | 100000325 | rragcb |  |  | 2747 | 1.097 | 0.1692 | No |
| 22 | 359839 | atp6v1ba |  |  | 2778 | 1.087 | 0.1743 | No |
| 23 | 359837 | wnt2ba |  |  | 2798 | 1.084 | 0.1798 | No |
| 24 | 100333504 | lamtor5 |  |  | 2872 | 1.065 | 0.1834 | No |
| 25 | 791993\_1 | wnt4b |  |  | 2954 | 1.048 | 0.1867 | No |
| 26 | 791563 | wnt16 |  |  | 3185 | 0.995 | 0.1851 | No |
| 27 | 557634 | si:ch211-147k10.5 |  |  | 3456 | 0.940 | 0.1818 | No |
| 28 | 58073 | fzd8a |  |  | 3761 | 0.876 | 0.1771 | No |
| 29 | 378866 | ddit4 |  |  | 3849 | 0.860 | 0.1792 | No |
| 30 | 777728 | rps6ka1 |  |  | 3888 | 0.850 | 0.1827 | No |
| 31 | 404211 | pik3r2 |  |  | 4059 | 0.817 | 0.1819 | No |
| 32 | 30105 | wnt5b |  |  | 4244 | 0.787 | 0.1805 | No |
| 33 | 792689 | mlst8 |  |  | 4430 | 0.756 | 0.1789 | No |
| 34 | 572074 | prkaa2 |  |  | 4710 | 0.717 | 0.1741 | No |
| 35 | 768159 | si:ch211-195b13.1 |  |  | 4819 | 0.702 | 0.1746 | No |
| 36 | 751630 | zgc:153713 |  |  | 4826 | 0.702 | 0.1784 | No |
| 37 | 100001198 | prkaa1 |  |  | 5040 | 0.675 | 0.1754 | No |
| 38 | 768177 | deptor |  |  | 5144 | 0.662 | 0.1759 | No |
| 39 | 562242\_1 | rps6kb1a |  |  | 5344 | 0.642 | 0.1732 | No |
| 40 | 100148840 | wnt7bb |  |  | 5854 | 0.602 | 0.1605 | No |
| 41 | 560638 | si:ch73-21k16.1 |  |  | 5961 | 0.591 | 0.1605 | No |
| 42 | 791595\_1 | wnt11 |  |  | 5972 | 0.590 | 0.1634 | No |
| 43 | 336619\_1 | eif4bb |  |  | 6245 | 0.563 | 0.1580 | No |
| 44 | 30171 | wnt10a |  |  | 6358 | 0.551 | 0.1575 | No |
| 45 | 557384 | fzd1 |  |  | 6390 | 0.548 | 0.1596 | No |
| 46 | 337583 | atp6v1ab |  |  | 6443 | 0.542 | 0.1610 | No |
| 47 | 337167\_2 | tsc1a |  |  | 6751 | 0.515 | 0.1542 | No |
| 48 | 30654 | gsk3b |  |  | 6859 | 0.504 | 0.1537 | No |
| 49 | 791563\_1 | wnt16 |  |  | 6996 | 0.492 | 0.1521 | No |
| 50 | 30127 | wnt2 |  |  | 7041 | 0.488 | 0.1535 | No |
| 51 | 558422 | lipin2 |  |  | 7174 | 0.475 | 0.1520 | No |
| 52 | 561737 | pik3ca |  |  | 7195 | 0.474 | 0.1540 | No |
| 53 | 751644 | wnt9a |  |  | 8426 | 0.408 | 0.1174 | No |
| 54 | 245701\_1 | igf1ra |  |  | 8653 | 0.391 | 0.1124 | No |
| 55 | 560455\_1 | rptor |  |  | 8701 | 0.388 | 0.1131 | No |
| 56 | 393750 | tbc1d7 |  |  | 9047 | 0.364 | 0.1043 | No |
| 57 | 768202 | pdpk1a |  |  | 9086 | 0.361 | 0.1051 | No |
| 58 | 324254 | mtor |  |  | 9164 | 0.355 | 0.1046 | No |
| 59 | 378972 | akt2 |  |  | 9293 | 0.347 | 0.1025 | No |
| 60 | 100329702 | ulk2 |  |  | 9563 | 0.329 | 0.0959 | No |
| 61 | 30366 | fzd10 |  |  | 9743 | 0.319 | 0.0920 | No |
| 62 | 415235 | cab39 |  |  | 9817 | 0.315 | 0.0915 | No |
| 63 | 337670 | rps6kal |  |  | 9943 | 0.307 | 0.0892 | No |
| 64 | 797213 | si:ch211-42i9.8 |  |  | 10175 | 0.292 | 0.0836 | No |
| 65 | 561835 | rictorb |  |  | 10438 | 0.276 | 0.0768 | No |
| 66 | 562137 | slc38a9 |  |  | 10616 | 0.265 | 0.0727 | No |
| 67 | 393228\_1 | zgc:55558 |  |  | 10754 | 0.257 | 0.0698 | No |
| 68 | 563181 | map2k2a |  |  | 10792 | 0.256 | 0.0701 | No |
| 69 | 101885386 | LOC101885386 |  |  | 10799 | 0.255 | 0.0713 | No |
| 70 | 30364 | fzd5 |  |  | 10873 | 0.250 | 0.0704 | No |
| 71 | 497384 | prkca |  |  | 10900 | 0.249 | 0.0710 | No |
| 72 | 245701 | igf1ra |  |  | 11103 | 0.239 | 0.0660 | No |
| 73 | 565714 | wnt7aa |  |  | 11249 | 0.231 | 0.0627 | No |
| 74 | 405785 | tnfa |  |  | 11720 | 0.205 | 0.0490 | No |
| 75 | 641489 | zgc:123305 |  |  | 11735 | 0.204 | 0.0497 | No |
| 76 | 399488 | slc3a2b |  |  | 11812 | 0.200 | 0.0484 | No |
| 77 | 368415 | ptenb |  |  | 11887 | 0.196 | 0.0472 | No |
| 78 | 393953 | prkcbb |  |  | 11901 | 0.196 | 0.0478 | No |
| 79 | 799566\_1 | eif4ebp1 |  |  | 12044 | 0.188 | 0.0444 | No |
| 80 | 100003514 | prkcg |  |  | 12176 | 0.181 | 0.0413 | No |
| 81 | 245700 | insrb |  |  | 12333 | 0.172 | 0.0373 | No |
| 82 | 100537790 | clip1b |  |  | 12441 | 0.167 | 0.0349 | No |
| 83 | 568518 | lrp5 |  |  | 12728 | 0.152 | 0.0267 | No |
| 84 | 492361 | cab39l |  |  | 12900 | 0.146 | 0.0221 | No |
| 85 | 100003877 | lrp6 |  |  | 12998 | 0.140 | 0.0198 | No |
| 86 | 103909433 | LOC103909433 |  |  | 13029 | 0.138 | 0.0196 | No |
| 87 | 561703 | rictora |  |  | 13067 | 0.137 | 0.0192 | No |
| 88 | 559391 | nprl2 |  |  | 13113 | 0.135 | 0.0186 | No |
| 89 | 569863 | dvl1a |  |  | 13418 | 0.120 | 0.0096 | No |
| 90 | 100170793 | ulk1a |  |  | 13675 | 0.108 | 0.0021 | No |
| 91 | 393533 | fzd6 |  |  | 13710 | 0.106 | 0.0017 | No |
| 92 | 103909113 | wdr59 |  |  | 13877 | 0.098 | -0.0030 | No |
| 93 | 327294 | gatsl2 |  |  | 14052 | 0.090 | -0.0080 | No |
| 94 | 561670 | sos2 |  |  | 14111 | 0.087 | -0.0094 | No |
| 95 | 493617 | eif4eb |  |  | 14120 | 0.086 | -0.0091 | No |
| 96 | 406336 | wdr24 |  |  | 14140 | 0.085 | -0.0093 | No |
| 97 | 553214 | eif4ba |  |  | 14146 | 0.085 | -0.0089 | No |
| 98 | 570568 | rragd |  |  | 14208 | 0.082 | -0.0104 | No |
| 99 | 100149794 | akt3b |  |  | 14249 | 0.080 | -0.0112 | No |
| 100 | 100003877\_1 | lrp6 |  |  | 14274 | 0.079 | -0.0115 | No |
| 101 | 30370 | fzd2 |  |  | 14334 | 0.077 | -0.0130 | No |
| 102 | 338219 | rps6ka3a |  |  | 14399 | 0.074 | -0.0146 | No |
| 103 | 245699 | insra |  |  | 14497 | 0.069 | -0.0173 | No |
| 104 | 794088\_1 | ptena |  |  | 15035 | 0.044 | -0.0340 | No |
| 105 | 559281 | irs1 |  |  | 15119 | 0.041 | -0.0364 | No |
| 106 | 562995 | depdc5 |  |  | 15249 | 0.035 | -0.0403 | No |
| 107 | 777634 | telo2 |  |  | 15269 | 0.034 | -0.0407 | No |
| 108 | 445155 | lamtor2 |  |  | 15308 | 0.032 | -0.0417 | No |
| 109 | 100537196 | mapkap1 |  |  | 15388 | 0.028 | -0.0440 | No |
| 110 | 30380 | nras |  |  | 15635 | 0.015 | -0.0517 | No |
| 111 | 569420 | wnt3 |  |  | 15807 | 0.006 | -0.0571 | No |
| 112 | 406308 | grb2b |  |  | 15876 | 0.003 | -0.0592 | No |
| 113 | 565921 | fzd3a |  |  | 15924 | 0.001 | -0.0607 | No |
| 114 | 797680 | clip1a |  |  | 15997 | -0.002 | -0.0630 | No |
| 115 | 557447 | fzd9a |  |  | 16011 | -0.002 | -0.0634 | No |
| 116 | 791141 | zgc:158659 |  |  | 16140 | -0.006 | -0.0674 | No |
| 117 | 324863 | fnip1 |  |  | 16210 | -0.008 | -0.0695 | No |
| 118 | 337167\_1 | tsc1a |  |  | 16372 | -0.013 | -0.0745 | No |
| 119 | 80972 | dvl3a |  |  | 16463 | -0.016 | -0.0773 | No |
| 120 | 550549 | eif4e1c |  |  | 16678 | -0.022 | -0.0839 | No |
| 121 | 570077 | tti1 |  |  | 16868 | -0.029 | -0.0897 | No |
| 122 | 541523 | eif4e2 |  |  | 17083 | -0.036 | -0.0963 | No |
| 123 | 100329656 | lamtor1 |  |  | 17121 | -0.037 | -0.0973 | No |
| 124 | 558848 | ulk1b |  |  | 17219 | -0.042 | -0.1001 | No |
| 125 | novel.839 | - |  |  | 17355 | -0.048 | -0.1041 | No |
| 126 | 30412 | fzd7b |  |  | 17559 | -0.056 | -0.1102 | No |
| 127 | 325358 | rraga |  |  | 17571 | -0.057 | -0.1102 | No |
| 128 | 406822\_1 | cab39l1 |  |  | 17580 | -0.057 | -0.1102 | No |
| 129 | 30363 | fzd4 |  |  | 17601 | -0.058 | -0.1105 | No |
| 130 | 560032 | dvl1b |  |  | 17603 | -0.058 | -0.1102 | No |
| 131 | 394174 | pik3cd |  |  | 17974 | -0.074 | -0.1214 | No |
| 132 | 100536950 | wu:fj77h11 |  |  | 17980 | -0.075 | -0.1212 | No |
| 133 | 550537 | stk11 |  |  | 18181 | -0.084 | -0.1270 | No |
| 134 | 406411 | rhoaa |  |  | 18330 | -0.091 | -0.1312 | No |
| 135 | 334749 | seh1l |  |  | 18500 | -0.099 | -0.1360 | No |
| 136 | 404211\_1 | pik3r2 |  |  | 18555 | -0.101 | -0.1371 | No |
| 137 | 445028 | rps6 |  |  | 18723 | -0.109 | -0.1418 | No |
| 138 | 567746 | fzd3b |  |  | 18726 | -0.110 | -0.1412 | No |
| 139 | 323630 | dvl2 |  |  | 19187 | -0.130 | -0.1551 | No |
| 140 | 767781 | rhebl1 |  |  | 19229 | -0.132 | -0.1556 | No |
| 141 | 560549 | akt3a |  |  | 19324 | -0.136 | -0.1578 | No |
| 142 | 399480 | mapk3 |  |  | 19514 | -0.144 | -0.1630 | No |
| 143 | 406728 | map2k1 |  |  | 19540 | -0.145 | -0.1630 | No |
| 144 | 565677 | wnt9b |  |  | 19640 | -0.150 | -0.1652 | No |
| 145 | 406349\_1 | rps6kb1b |  |  | 19701 | -0.153 | -0.1663 | No |
| 146 | 797250\_1 | slc7a5 |  |  | 19759 | -0.155 | -0.1672 | No |
| 147 | 245701\_2 | igf1ra |  |  | 20190 | -0.174 | -0.1798 | No |
| 148 | 557109 | raf1b |  |  | 20203 | -0.175 | -0.1792 | No |
| 149 | 406471 | tnfrsf1a |  |  | 20265 | -0.178 | -0.1802 | No |
| 150 | 493609\_1 | grb2a |  |  | 20689 | -0.198 | -0.1924 | No |
| 151 | 564989 | nprl3 |  |  | 20758 | -0.202 | -0.1934 | No |
| 152 | 393208 | mios |  |  | 20762 | -0.202 | -0.1924 | No |
| 153 | 30128 | wnt1 |  |  | 21411 | -0.236 | -0.2116 | No |
| 154 | 566559 | akt1s1 |  |  | 21628 | -0.247 | -0.2170 | No |
| 155 | 541544 | rnf152 |  |  | 21689 | -0.251 | -0.2175 | No |
| 156 | 30283 | wnt11r |  |  | 21916 | -0.263 | -0.2232 | No |
| 157 | 559050 | sgk2b |  |  | 22037 | -0.269 | -0.2254 | No |
| 158 | 245701\_3 | igf1ra |  |  | 22143 | -0.275 | -0.2272 | No |
| 159 | 334550 | strada |  |  | 22278 | -0.282 | -0.2299 | No |
| 160 | 566735 | insb |  |  | 22312 | -0.285 | -0.2293 | No |
| 161 | 100332722 | wnt6a |  |  | 22571 | -0.300 | -0.2358 | No |
| 162 | 324254\_1 | mtor |  |  | 22584 | -0.301 | -0.2345 | No |
| 163 | 403065 | braf |  |  | 22710 | -0.308 | -0.2367 | No |
| 164 | 553597 | lamtor3 |  |  | 22898 | -0.319 | -0.2408 | No |
| 165 | 30144 | wnt8b |  |  | 23062 | -0.332 | -0.2441 | No |
| 166 | 406659 | rhoac |  |  | 23154 | -0.338 | -0.2451 | No |
| 167 | 108179059 | LOC108179059 |  |  | 23224 | -0.343 | -0.2454 | No |
| 168 | 100151344 | si:dkeyp-97e7.9 |  |  | 23343 | -0.350 | -0.2471 | No |
| 169 | 360144\_1 | mapk1 |  |  | 23446 | -0.357 | -0.2484 | No |
| 170 | 335025 | atp6v1g1 |  |  | 23477 | -0.359 | -0.2473 | No |
| 171 | 100002263 | pik3cb |  |  | 23508 | -0.361 | -0.2462 | No |
| 172 | 557882 | si:dkeyp-80c12.7 |  |  | 23526 | -0.363 | -0.2447 | No |
| 173 | 557176 | pik3r1 |  |  | 23610 | -0.367 | -0.2453 | No |
| 174 | 561019 | lamtor4 |  |  | 23651 | -0.371 | -0.2445 | No |
| 175 | 563427 | wnt7ba |  |  | 23760 | -0.377 | -0.2458 | No |
| 176 | 393732 | eif4e2rs1 |  |  | 24066 | -0.397 | -0.2532 | No |
| 177 | 561831 | wnt6b |  |  | 24096 | -0.399 | -0.2519 | No |
| 178 | 393935 | atp6v1d |  |  | 24235 | -0.409 | -0.2539 | No |
| 179 | 394110\_1 | atp6v1aa |  |  | 24258 | -0.411 | -0.2523 | No |
| 180 | 678628 | flcn |  |  | 24345 | -0.415 | -0.2527 | No |
| 181 | 337583\_1 | atp6v1ab |  |  | 24458 | -0.417 | -0.2539 | No |
| 182 | 100002225 | map2k2b |  |  | 24736 | -0.433 | -0.2602 | No |
| 183 | 100006041 | rhoab |  |  | 24773 | -0.436 | -0.2589 | No |
| 184 | 406822 | cab39l1 |  |  | 24846 | -0.440 | -0.2587 | No |
| 185 | 393287 | chuk |  |  | 25038 | -0.449 | -0.2623 | No |
| 186 | 100538220 | atp6v1c2 |  |  | 25059 | -0.451 | -0.2604 | No |
| 187 | 100537865 | prr5b |  |  | 25074 | -0.452 | -0.2583 | No |
| 188 | 100534937 | LOC100534937 |  |  | 25301 | -0.467 | -0.2628 | No |
| 189 | 449655 | atp6v1c1b |  |  | 25334 | -0.469 | -0.2612 | No |
| 190 | 100149745 | sesn2 |  |  | 25398 | -0.475 | -0.2605 | No |
| 191 | 568191 | wnt5a |  |  | 25472 | -0.482 | -0.2601 | No |
| 192 | 562032 | grb10b |  |  | 25659 | -0.499 | -0.2632 | Yes |
| 193 | 568110 | LOC568110 |  |  | 25683 | -0.501 | -0.2611 | Yes |
| 194 | 359839\_1 | atp6v1ba |  |  | 25690 | -0.502 | -0.2585 | Yes |
| 195 | 790938\_1 | zgc:158423 |  |  | 25805 | -0.511 | -0.2592 | Yes |
| 196 | 30716 | raf1a |  |  | 25882 | -0.518 | -0.2587 | Yes |
| 197 | 436799 | atp6v1f |  |  | 25896 | -0.520 | -0.2562 | Yes |
| 198 | 30308 | wnt10b |  |  | 26044 | -0.533 | -0.2579 | Yes |
| 199 | 403000 | pdpk1b |  |  | 26060 | -0.534 | -0.2554 | Yes |
| 200 | 192335 | atp6v1e1b |  |  | 26172 | -0.545 | -0.2558 | Yes |
| 201 | 286779 | atp6v1h |  |  | 26354 | -0.562 | -0.2584 | Yes |
| 202 | 550286 | hrasa |  |  | 26472 | -0.575 | -0.2589 | Yes |
| 203 | 100333967 | si:ch73-374l24.1 |  |  | 26564 | -0.584 | -0.2585 | Yes |
| 204 | 335025\_1 | atp6v1g1 |  |  | 26589 | -0.587 | -0.2560 | Yes |
| 205 | 567524 | tsc2 |  |  | 26670 | -0.595 | -0.2551 | Yes |
| 206 | 100149082 | dvl3 |  |  | 26821 | -0.611 | -0.2565 | Yes |
| 207 | 569420\_1 | wnt3 |  |  | 26844 | -0.613 | -0.2537 | Yes |
| 208 | 559391\_1 | nprl2 |  |  | 26970 | -0.627 | -0.2542 | Yes |
| 209 | 114433 | igf1 |  |  | 27128 | -0.641 | -0.2555 | Yes |
| 210 | 30738 | eif4e1b |  |  | 27314 | -0.650 | -0.2577 | Yes |
| 211 | 30123 | wnt4a |  |  | 27492 | -0.672 | -0.2596 | Yes |
| 212 | 449937 | gatsl3 |  |  | 27540 | -0.677 | -0.2572 | Yes |
| 213 | 553656 | hrasb |  |  | 27554 | -0.680 | -0.2538 | Yes |
| 214 | 560455 | rptor |  |  | 27664 | -0.695 | -0.2534 | Yes |
| 215 | 30262 | ins |  |  | 27693 | -0.700 | -0.2504 | Yes |
| 216 | 794088\_2 | ptena |  |  | 27793 | -0.715 | -0.2495 | Yes |
| 217 | 497384\_1 | prkca |  |  | 27854 | -0.723 | -0.2473 | Yes |
| 218 | 678628\_1 | flcn |  |  | 27924 | -0.731 | -0.2454 | Yes |
| 219 | 60632 | wnt3a |  |  | 28059 | -0.750 | -0.2454 | Yes |
| 220 | 799099 | lpin1 |  |  | 28128 | -0.760 | -0.2433 | Yes |
| 221 | 101910198 | akt1 |  |  | 28441 | -0.810 | -0.2487 | Yes |
| 222 | 368907 | atp6v1c1a |  |  | 28719 | -0.863 | -0.2526 | Yes |
| 223 | 798071\_1 | sec13 |  |  | 28754 | -0.869 | -0.2488 | Yes |
| 224 | 796322\_1 | slc3a2a |  |  | 28952 | -0.909 | -0.2499 | Yes |
| 225 | 393702 | rheb |  |  | 29061 | -0.934 | -0.2481 | Yes |
| 226 | 445289 | kras |  |  | 29269 | -0.994 | -0.2491 | Yes |
| 227 | 403146 | akt2l |  |  | 29342 | -1.012 | -0.2457 | Yes |
| 228 | 571285 | rps6ka2 |  |  | 29423 | -1.039 | -0.2424 | Yes |
| 229 | 556286 | sos1 |  |  | 29488 | -1.060 | -0.2384 | Yes |
| 230 | 100009657 | rps6ka3b |  |  | 29693 | -1.126 | -0.2386 | Yes |
| 231 | 797250 | slc7a5 |  |  | 29886 | -1.197 | -0.2379 | Yes |
| 232 | 100006675\_1 | prkcba |  |  | 30078 | -1.276 | -0.2368 | Yes |
| 233 | 799566 | eif4ebp1 |  |  | 30170 | -1.323 | -0.2323 | Yes |
| 234 | 563708 | skp2 |  |  | 30286 | -1.382 | -0.2282 | Yes |
| 235 | 791993 | wnt4b |  |  | 30739 | -1.714 | -0.2329 | Yes |
| 236 | 79380\_1 | eif4ea |  |  | 30785 | -1.745 | -0.2245 | Yes |
| 237 | 30369\_1 | fzd7a |  |  | 30807 | -1.766 | -0.2153 | Yes |
| 238 | 30127\_1 | wnt2 |  |  | 30890 | -1.852 | -0.2075 | Yes |
| 239 | 573492 | rragca |  |  | 31042 | -2.018 | -0.2010 | Yes |
| 240 | 359840\_1 | atp6v1b2 |  |  | 31211 | -2.252 | -0.1936 | Yes |
| 241 | 245702\_1 | igf1rb |  |  | 31247 | -2.324 | -0.1817 | Yes |
| 242 | 100000252\_1 | pik3r3b |  |  | 31434 | -2.733 | -0.1723 | Yes |
| 243 | 792689\_1 | mlst8 |  |  | 31444 | -2.758 | -0.1571 | Yes |
| 244 | 58072 | fzd8b |  |  | 31520 | -2.978 | -0.1428 | Yes |
| 245 | 555584 | prr5a |  |  | 31549 | -3.054 | -0.1266 | Yes |
| 246 | 336619 | eif4bb |  |  | 31594 | -3.240 | -0.1098 | Yes |
| 247 | 556087\_1 | wnt2bb |  |  | 31672 | -3.576 | -0.0922 | Yes |
| 248 | 790938 | zgc:158423 |  |  | 31716 | -3.826 | -0.0721 | Yes |
| 249 | 436718\_1 | rhocb |  |  | 31770 | -4.295 | -0.0497 | Yes |
| 250 | 324140 | sgk1 |  |  | 31816 | -4.750 | -0.0245 | Yes |
| 251 | 100333504\_1 | lamtor5 |  |  | 31823 | -4.786 | 0.0021 | Yes |
Table: GSEA details [plain text format]

  

Fig 2: MTOR\_SIGNALING\_PATHWAY(DRE04150)      
 Blue-Pink O' Gram in the Space of the Analyzed GeneSet

  

Fig 3: MTOR\_SIGNALING\_PATHWAY(DRE04150): Random ES distribution      
 Gene set null distribution of ES for **MTOR\_SIGNALING\_PATHWAY(DRE04150)**
